# Burnout and Attention Failure in STEM: The Role of Self-Control and the Buffer of Mindfulness

**DOI:** 10.3390/ijerph21081000

**Published:** 2024-07-30

**Authors:** Mahima Saxena

**Affiliations:** Industrial-Organizational Psychology, Department of Psychology, University of Nebraska Omaha, 6001 Dodge St. ASH 347, Omaha, NE 68182, USA; msaxena@unomaha.edu

**Keywords:** burnout, mindfulness, STEM, mind-wandering, Johnson-Neyman, attention

## Abstract

Drawing on self-regulatory strength models of self-control, this research examined the relationship between burnout and attentional processes for STEM (science, technology, engineering, and mathematics) students. Using data from participants in STEM, burnout was found to be associated with higher levels of off-task thinking, also known as mind-wandering. Further, self-control acted as a mediator in the relationship between burnout and mind-wandering such that higher levels of burnout predicted poor self-control that subsequently increased the mind’s tendency to wander. Additionally, mindfulness buffered the relationship between burnout and mind-wandering such that burnout had the most detrimental impact on attention for those students who were low in mindfulness. Using the Johnson-Neyman approach, results reveal the upper limits for the moderating impact of mindfulness. Results and implications for science and practice are discussed with a special grounding for students in the STEM context.

## 1. Introduction

Burnout has been researched in psychological sciences since the early 1990’s. Widely understood as a chronic depletion of internal energetic resources [1,2], burnout is known to be associated with a wide range of negative outcomes for organizations and universities. For employees suffering with job-burnout, negative outcomes include, most prominently, lower performance [3,4,5], reduction in extra-role behaviors [6], poor work-related attitudes (lower job-satisfaction, organizational commitment, and turnover intentions [7,8]), and negative interpersonal relationships both in and out of the workplace [9,10].

Similarly, academic burnout is associated with poor classroom performance [11], reduction in extra-curricular activities [12], and reduced motivation [13]. From a health and well-being perspective, burnout is associated with chronic inflammatory markers [14], gastro-intestinal upsets [15], cardiovascular incidence [16], and psychological symptoms that mimic depression and chronic fatigue syndrome [17,18]. Studies have also shown that higher levels of academic burnout are associated with increased cardiovascular risk [19]. Overall, regardless of the context in which it occurs (school, work, and non-work), there is no dearth of literature pointing to the myriad detrimental consequences of burnout.

A widely studied outcome of burnout is the negative impact it has on performance [20]. Burnout is associated with declines in performance. Burnt-out individuals tend to report reduced interest, poor motivation, and diminished performance across domains as wide as education [11], nursing [21], customer care [22], teaching [23], and management [24].

Attention focus is a core aspect of task-performance and can help further elucidate burnout’s impact on the cognitive basis of behavior [25]. Using depletion and self-regulatory strength models of self-control, this study examines burnout’s impact on attention via mind-wandering for those in STEM. Specifically, it is proposed that the chronic depletion of internal energetic resources in burnout leads to difficulty in maintaining attentional focus. Further, this connection is explained through the reduction in self-control capacities. This in turn manifests in an inability to focus attention on tasks and may subsequently lead to performance decrements that have been documented in extant research. Additionally, mindfulness is examined as an individual difference trait that can buffer the negative impact of burnout on attention-failure by maintaining mental awareness in the present moment and allowing individuals to counter the negative impact of burnout on attentional systems.

It has been well documented that individuals across multiple contexts and domains experience diminished performance in the face of burnout [3]. This has been observed in the case of caregiver burnout [26], athletes [27], teachers [28], students [28], medical professionals including those in nursing [29], physicians [30], and surgeons, and in the customer service industry [31]. These performance decrements have significant near-term and long-term consequences for those suffering from burnout. As a distal consequence, these could include threats to workers’ livelihoods as diminished performance in the context of work may pose a risk to employment via poor performance ratings, poor organizational appraisals, and, ultimately, layovers [32]. As a proximal consequence, poor performance tends to be associated with reduced self-efficacy, disinterest, and lack of motivation [33]. In the academic or school setting, students suffering from poor academic performance are at risk of poor grades, lower self-esteem, lack of self-efficacy about their education, and, eventually, adverse consequences such as dropping out of college with significant negative outcomes for students’ future careers and lives [34].

From a scientific standpoint, investigating how burnout leads to such decrements in performance is critical to developing a nuanced and in-depth understanding of the nature and consequences of this rapidly increasing public health problem. Importantly, such an understanding may potentially lead to important interventions that may be useful for preventing such adverse outcomes from occurring.

In general, while the extant literature documents the ill-effects of burnout on performance related outcomes [20,35], there is relatively less research on the inner cognitive processes that may drive these reductions in performance. For instance, it was noted that there is “sparse but relatively consistent evidence” [36] suggesting that exhaustion in burnout adversely impacts cognitive functioning. Beal et al. [25] purported that the fundamental substrate of performance is attention. In other words, the primary building block of performance is attention. Attentional mechanisms rely on underlying pools of resources that essentially drive the attention process. Thus, the study of attention is key in the study of burnout. The current study aims to fill this gap by focusing on the cognitive consequences and inner psychological drivers that may help explain why burnout has adverse effects on performance. Specifically, it investigates mind-wandering as a core attentional process. An additional strength of the present study is that it does so in field settings, outside of the lab in a real-world sample of individuals that may be prone to burnout.

## 2. Theoretical Basis and Hypothesis Development

### 2.1. Chronic Self-Regulatory Depletion in Burnout

Burnout is, “an affective reaction to ongoing stress whose core content is the gradual depletion over time of individuals’ intrinsic energetic resources, including emotional exhaustion, physical fatigue, and cognitive weariness” [1]. Burnout is understood as a chronic response to a long period of exposure to stressful and demanding circumstances, such that it operates enduringly and long-term. Unlike stress, anxiety, and emotion, which are states [37], burnout is chronic. The regulatory strength model [38,39] helps us understand the psychological underpinnings of burnout. This study conceptualizes burnout as a long-term depletion of internal energetic resources [2], a chronic condition wherein the individual has “run out of” resources that are necessary for efficient living and functioning. This state of fatigue is particularly conspicuous in higher order goal-oriented cognitive activities such as working memory [40] and mind-wandering [41]. Thus, burnout is conceptualized as characterized by chronic depletion, where the individual has completely spent the resources necessary to engage in regulation of the self [42]. In other words, burnout takes away the ability to draw from an internal pool of resources, simply because there are no more resources left in this state of chronic depletion [2,43,44], thereby making any effortful self-regulation unsuccessful. This is why burnt-out individuals experience cynicism, withdrawal, reduced self-efficacy, and are unable to engage emotionally with others [1,45]—all phenomena that are effortful and rely on an internal pool of energy.

### 2.2. Mind-Wandering

The mind has the fundamental property of wandering. These include attentional biases, distractions, memory lapses, ruminating thoughts, and various other types of disruptive thoughts and thought patterns [46]. The experience of thoughts that are unrelated to the task at hand occur due to various internal and external reasons and can act as an obstruction to ongoing activity by diverting attention away from the primary task. These include worry [47], anxiety [48,49], external and internal triggers [50,51], and fatigue [41], among others.

Underlying regulatory control is essential to impede “other” thoughts from popping into the mind and prevent it from wandering. Due to the chronic depletion of underlying energetic resources, burnout is marked by limited energy [52] to successfully manage mental activity. This is corroborated by evidence indicating that burnout is marked by the experience of cognitive weariness and self-reported deficits in basic cognitive processes such as memory and perception [1,53]. Given burnout occurs when an individual is chronically depleted, the lack of intrinsic resources will prevent an individual from maintaining the stream of thought on task-relevant activity, and such individuals will be more prone to the distraction of intrusive thought, thereby increasing attention-failure. In the presence of burnout, individuals will experience higher levels of mind-wandering. Thus, the following hypothesis is proposed:

**Hypothesis 1:**  *Burnout is positively associated with mind-wandering.*

### 2.3. The Role of Self-Control

Self-control ability has received attention recently as an explanatory mechanism for the deleterious effects of burnout [54]. Self-control is the ability of the self to exercise restraint over oneself, alter inner experiences, and change, override, or modify behaviors such as impulses and temptations [55]. Self-control relies on both dispositional and state characteristics that may vary between individuals [56,57]. Trait self-control behaves as an individual difference variable, similar to personality, such that it is less susceptible to change and remains relatively stable over time. This trait or dispositional self-control is generally assessed using self-report measures and has been associated with academic performance, psychopathology, and physical and verbal aggression [54,55]. On the other hand, state self-control may fluctuate over the course of the day and tends to be more fluid in nature [56,58]. Research traditions in state self-control generally engage in experimental manipulation through a task of self-regulatory depletion and examine the effects on future acts of self-control [41].

Within academic settings, using two separate samples in a cross-sectional study, Siebert et al. [54] found a negative relationship between school burnout and dispositional self-control. In other words, participants who experienced burnout demonstrated lower levels of dispositional self-control. The link between burnout and self-control may be explained using the self-regulatory strength model [59] and in their relationship with trait differences in executive functions [60]. First, as conceptualized previously, burnout is marked by chronic depletion of internal resources. Self-regulatory strength models of self-control and evidence from subsequent research suggest that self-control operates on the basis of an underlying pool of limited and finite resources that allow an individual to exercise control and modify behaviors and inner states (such as attention, emotion, and other subjective phenomena [59]). As the underlying resource becomes depleted, the ability to exercise self-control becomes less effective, thereby leading to adverse outcomes. Given the lack of internal resources from which one can draw energy, acts of self-control may potentially be undermined due to the presence of burnout. Thus, based on the self-regulatory strength model, it can be surmised that burnout may lead to reduction in self-control due to the lack of internal resources which provide the energy to engage in self-control ability. Thus, self-control may diminish in response to depleted resources.

Further support for this theorizing comes from research in executive functions. According to a unified, integrated model of self-control, it has been suggested that trait differences in executive functioning influence the exhaustion of resources [60]. Executive functions, in turn, influence self-control, and both are reliant on an underlying shared depletable resource [61,62]. School burnout impairs executive functioning, demonstrated by poor performance on cognitive tasks [19], and on trait self-control leading to poor academic performance 36. This relationship has yet to be examined in STEM settings.

While the mind has the fundamental property of throwing up thoughts unrelated to present reality, self-control is the mechanism by which such interfering or distracting thoughts can be curtailed. In other words, effortful self-control can suppress interfering thoughts in the face of distraction to maintain attentional focus. However, the reduction in self-control due to depleted resources in burnout will influence the ability to curtail mind-wandering. This will manifest in increased distracting thoughts. In other words, self-control will mediate the burnout and mind-wandering link such that burnout will have a negative relationship with self-control, which, in turn, will have a negative relationship with attention-failure. Thus, burnout will be associated with reduced self-control, which, in turn, will be associated with higher levels of mind-wandering. Overall, based on the above, the following is proposed:

**Hypothesis 2a:**  *Burnout is negatively associated with self-control.*

**Hypothesis 2b:**  *Self-control is negatively associated with mind-wandering.*

**Hypothesis 2c:**  *Burnout is indirectly associated with mind-wandering through the mechanism of self-control.*

### 2.4. The Role of Mindfulness

Mindfulness is understood in Western literature as the ability to stay in the present moment, grounded in the current reality, without judgment or psychological fusion to passing thoughts, emotions, or other inner experiential states [63,64]. Mindfulness has been conceptualized as a state [65] and as a trait in terms of the predisposition to be mindful in daily life [66,67]. It has been suggested that mindfulness is an innate human trait, amenable to changes through training and practice [68]. Indeed, mindfulness training appears to have a positive impact on affect regulation, reduction in stress, higher levels of self-reported well-being [69,70,71], and improved performance on tasks of working memory and reading comprehension [72]. Some studies have found that mindfulness may be able to increase resources to prevent school burnout [73].

Research on mindfulness as a disposition describes it as an ability of individuals to stay grounded in their present realities. As an individual difference factor, mindfulness reflects the innate capacity to be mindful and be aware of what is occurring in the present and to simply observe reality as it unfolds [63]. By staying grounded in the present moment, individuals would be able to avoid the distraction of mental interference: the intrusive thoughts that may otherwise undermine immersion in the present moment. This focus on the present moment would therefore serve as a buffer against the wandering mind in the face of burnout. In other words, despite burnout, individuals higher in mindfulness would be able to resist the onslaught of wandering thoughts given their innate ability to stay grounded in the present moment. Overall, it is proposed that mindfulness would serve as a buffer against the detrimental impact of burnout on attention failure. Therefore, the following is hypothesized:

**Hypothesis 3:**  *The direct association between burnout and mind-wandering is conditional upon the influence of mindfulness such that those with higher levels of mindfulness will have the weakest direct link.*

**Figure 1:**
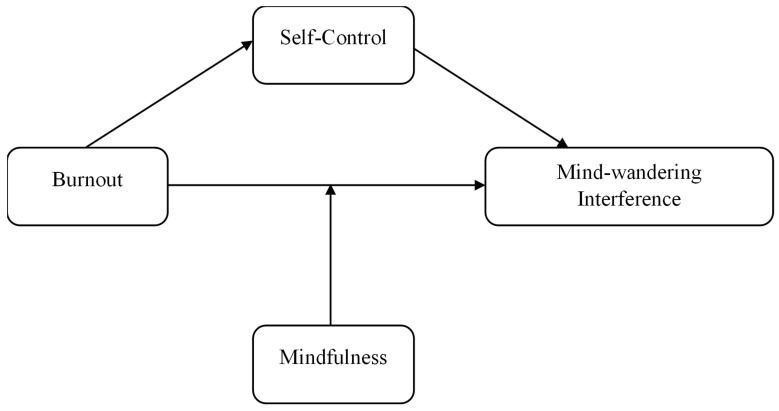
Conceptual model with all study variables.

Overall, by examining the harmful effects of burnout on attentional focus through the mediation of self-control, this study can help understand potential pathways and mechanisms for how and why burnout may have a negative effect on performance across various settings including academia and industry. The full conceptual model with all study variables is displayed in Figure 1.

### 2.5. The STEM Context of the Present Investigation

The present investigation was conducted with the backdrop of science, technology, engineering, and mathematics (STEM) fields in education. STEM fields in general tend to be characterized by highly demanding work environments [74], often marked by fast deadlines, immersive work, competition [75], pressure to succeed [76], and high rates of rejection in associated academia and industry [77]. STEM students are likely to suffer from high levels of anxiety, burnout, disengagement [78], and impaired psychological well-being [74]. Similar characteristics are found in STEM workplaces [79]. Thus, the STEM backdrop is considered an appropriate context for the present study, for purposes of ecological validity and for practical implications that may emerge given the findings of this investigation.

## 3. Methods

### 3.1. Ethics Approval

This study was approved by the university’s Institutional Review Board and was performed in accordance with relevant guidelines and regulations. All participants provided informed consent prior to participation.

### 3.2. Participants and Procedure

Data were collected from a medium-sized Midwestern educational institution in the United States of America. Data were collected over the period of an academic semester. Participants were full-time students enrolled in science, technology, engineering, and math, above the age of 18, recruited through various forms of advertisement in university settings. These included word-of-mouth sampling, advertising around the university, in-class recruitment, and in common areas where students tended to collect in their free time. Professors offered this study as one of the options for extra credit through an online participant management system. Participants were requested to fill out an online survey on college student experiences and wellbeing. They were requested to respond with the first thought that came to mind in response to the survey questions. Participants were assured of the confidential, anonymous, and academic nature of this study to mitigate potential biases that may arise due to social desirability. A total of 513 students completed an online survey. The average age of the sample was 21 years (SD = 4.12). Students were STEM majors in a variety of STEM disciplines including engineering, computer sciences, biology, chemistry, physics, and architecture. The majority of students were engineering majors (46%), followed by computer science (13%), information technology and management (10%), architecture (8%), and the remaining were distributed across biology, chemistry, physics, psychological sciences, and so on. Students were distributed across all years of college education, with the overwhelming majority (76%) in the first three years of their bachelors’ studies (freshman = 22%, sophomore = 28%, junior = 26%, senior = 18%, and the remaining in other advanced years). The sample was roughly 59% male (40% female, 1% other), and represented the following ethnicities: 42% Caucasian, 28% Asian, 13% Hispanic, and about 7% African-American, and the remaining indicated mixed race or others.

### 3.3. Measures

#### 3.3.1. Burnout

Burnout was assessed using the 15-item academic version of the Maslach Burnout Inventory [12]. Each item was assessed on a five-point scale from 1 (never to almost never) to 7 (always or almost always). Example items include “I feel burned out from my studies”, “I feel tired when I get up in the morning and I must face another day at the university”, “I doubt the significance of my studies”, and “I have become less enthusiastic about my studies”. This scale is widely used to assess burnout in academic settings. Cronbach’s coefficient alpha in the current study was 0.90, which indicates good reliability of this measure in this study.

#### 3.3.2. Self-Control

Trait self-control was assessed using the 36-item Self-control Scale [55]. Items were rated on a five-point scale ranging from 1 (not accurately at all) to 5 (extremely accurately). Items required participants to rate how accurately each prompt described them. Example item are “I blurt out whatever is on my mind”, “I have a hard time breaking bad habits”, “I wish I had more self-discipline”, and “I often interrupt people”. Cronbach’s co-efficient alpha was 0.89 in the current study, suggesting good reliability of the measure in the current study.

#### 3.3.3. Mind-Wandering

The mind-wandering subscale from the Imaginal Process Inventory (IPI [80]) was used to assess mind-wandering. Of the two traditions in mind-wandering assessment, i.e., thought-probes with or without the use of laboratory-based experimental tasks [81] and questionnaire assessment [82,83], the IPI is the oldest, psychometrically sound, and most widely utilized self-report instrument to measure mind-wandering [81,84]. The original reliability index ranges between 0.70 and 0.91. A total of 12 items were assessed on a five-point scale from 1 (Definitely not true for me) to 5 (Very true for me). An example item is “My mind seldom wanders while I am working”, “I have difficulty maintaining concentration for long periods of time”, and “No matter how hard I try to concentrate, thoughts unrelated to my work always creep in”. The coefficient alpha in the current study was 0.79, indicating suitable Cronbach’s internal consistency reliability.

#### 3.3.4. Mindfulness

The 15-item Mindful Attention Awareness Scale [68] was used to assess dispositional mindfulness. Example items are “I forget a person’s name almost as soon as I’ve been told it for the first time”, “I find myself preoccupied with the future or the past”, and “I snack without being aware that I’m eating”, rated on a scale of 1 (almost always) to 6 (almost never). Cronbach’s co-efficient alpha was 0.90 in the current study, suggesting suitable reliability for the purposes of the current study.

## 4. Results

Means, standard deviations, reliabilities, and correlations for all study variables are reported in Table 1. Hypothesis testing results are presented in Table 2. The figures present the hypothesis testing results.

### 4.1. Analytical Strategy

Three successive analyses were conducted to test the hypotheses. Specifically, first, an OLS regression was conducted to examine the association of burnout and mind-wandering (Hypothesis 1). All remaining hypotheses were tested using the PROCESS macro for SPSS [85,86,87]. Second, following recommendations of Preacher and Hayes [88], Shrout and Bolger [89], and Hayes [85], SPSS PROCESS macros, version 3.2.02, were used to examine the overall mediated model using a bias-corrected bootstrapping procedure [85]. In other words, the indirect effect of burnout on mind-wandering through self-control was assessed using bootstrapping in Model 1 (Hypotheses 2a, 2b, 2c [85,90]). Finally, a combined conditional process model using bootstrapping was utilized to assess the moderator effect of mindfulness on the relationship between burnout and mind-wandering. Model 5 [85] was utilized to simultaneously test for the indirect effect of burnout on mind-wandering through the mediating mechanism of self-control, as well as the moderation of the direct effect of burnout on mind-wandering by mindfulness.

Bootstrapping involved resampling with replacement from the data set, yielding a sampling distribution of the indirect effect. Bootstrapped confidence intervals were computed using 5000 replications. This was used to construct confidence intervals with 95% significance. Confidence intervals that exclude zero provide evidence of significant effects [89]. Bootstrapping procedures are advantageous since they allow the researcher to work with non-normal sampling distributions and permit greater statistical power while reducing the likelihood of Type 1 error [88,91]. To provide a formal test for moderation, the index of moderation was examined, which implies that any two conditional effects defined by different values of the moderator are statistically different [88]. 

All path coefficients are reported as unstandardized OLS regression coefficients following recommendations for preferred metrics in causal modeling [92,93,94]. Product terms were mean centered to better interpret direct effects in the moderation model. The pattern of results was the same with and without the inclusion of control variables (age, sex, year in the program). Therefore, per recommendations from the existing literature, the reported results exclude current variables [95,96].

### 4.2. Hypothesis Testing

**Hypothesis 1.** Hypothesis 1 predicted that burnout is positively associated with mind-wandering. Results from an OLS regression model reveal a significant and positive association between burnout and mind-wandering (total effect, *c* = 0.26, *p* < 0.001).

**Hypothesis 2a, 2b, and 2c.** Hypothesis 2 predicted that burnout is negatively associated with self-control (Hypothesis 2a), self-control is negatively associated with mind-wandering (Hypothesis 2b), and that burnout is indirectly associated with mind-wandering through the mediating mechanism of self-control (Hypothesis 2c). Results from Model 4 in PROCESS suggest that burnout has a significant negative association with self-control (*a* effect = −0.23, *p* < 0.001). Hypothesis 2a was supported. Self-control had a significant negative association with mind-wandering (*b* effect = −0.38, *p* < 0.001). Hypothesis 2b was supported. Finally, burnout exerted a significant indirect effect on mind-wandering through self-control (*indirect effect* = 0.09, CI_.95_ = 0.059, 0.118). The bias-corrected confidence interval did not contain 0, providing support for Hypothesis 2b. Independent of this mediation pathway, the remaining direct effect of burnout on mind-wandering was also significant (*c’ effect* = 0.17, *p* < 0.001). Overall, the total effect of burnout on mind-wandering (*c effect*) was a function of the indirect effect of (a*b) and the remaining direct effect (*c’*).

**Hypothesis 3.** Hypothesis 3 predicted that the direct association between burnout and mind-wandering is conditional upon the influence of mindfulness such that those with higher levels of mindfulness have a weaker association between burnout and mind-wandering. Results from PROCESS Model 5 reveal that mindfulness moderated the direct relationship between burnout and mind-wandering. The index of moderation b3 (*b3* = −0.05, CI_.95_ = −0.091, −0.006) was significantly different from 0. Thus, mindfulness acts as a moderator of the direct effect.

### 4.3. Probing the Interaction

**Figure 2:**
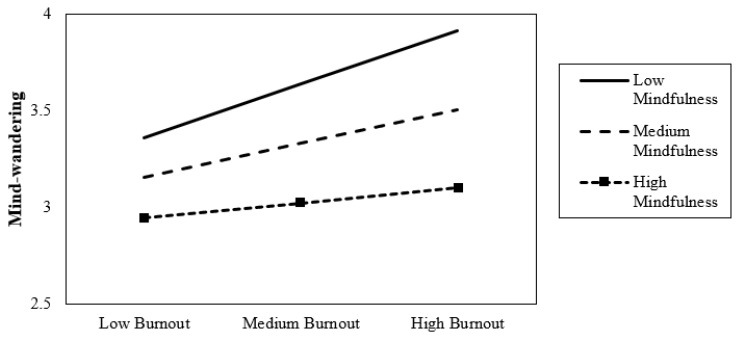
The direct effect of burnout on mind-wandering moderated by mindfulness. *Note:* Level of mindfulness is 16th, 50th and 84th percentile.

To interpret the pattern of this conditional effect, we visualized the effects using values for the 16th, 50th, and 84th percentiles for mindfulness. These values were specifically chosen in order to not extrapolate beyond the available data [85]. The resulting pattern of results can be seen in Figure 2. As expected, the effect of burnout on mind-wandering was conditional upon the level of mindfulness. The positive association between burnout and mind-wandering was stronger under relatively low (Ø*_X→Y|W_*_=−0.793_ = 0.174, *p* < 0.001), medium under moderate (Ø*_X→Y|W_*_=0.007_ = 0.135, *p* < 0.001) and weakest under high (Ø*_X→Y|W_*_=0.873_ = 0.093, *p* < 0.05) levels of mindfulness. All slopes are positive; however, the most positive slope emerges when the level of mindfulness is the lowest. Thus, the direct effect becomes increasingly positive as mindfulness decreases. Overall, at higher levels of mindfulness, the relationship between burnout and mind-wandering becomes weaker than for lower levels of mindfulness.

**Figure 3:**
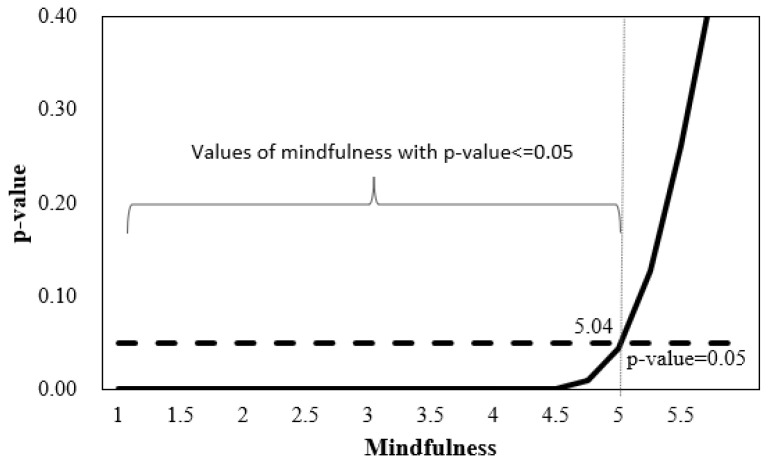
Johnson-Neiman region of significance for the conditional effects. *Note:* 92% of the values of mindfulness < 5.04.

**Figure 4:**
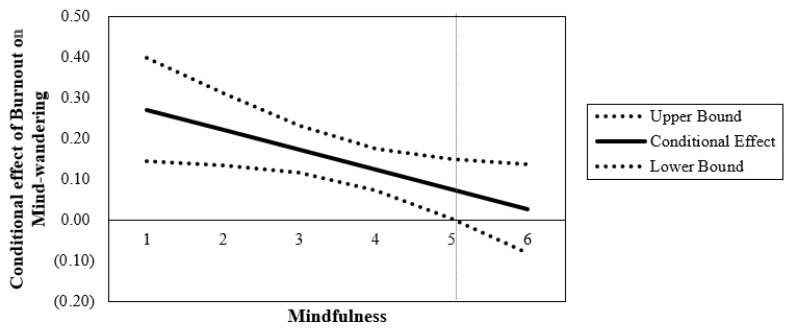
Conditional direct effect of burnout with confidence bands. *Note:* Bands indicate 95% confidence for conditional effect of burnout on mind-wandering. Vertical line denotes the value of this effect at 5.04.

To further understand how burnout’s effect changes as a function of mindfulness, the Johnson-Neyman (JN) technique was utilized [97,98]. This floodlight analysis mathematically derived the point on the continuum of the moderator variable (mindfulness) where the effect of burnout transitions from statistically significant to statistically non-significant. The resulting graphs are depicted in Figure 3 and Figure 4. The centered moderator value defining the Johnson-Neyman region of significance is *W* = 1.243, such that approximately 92% of the data is below this value. In other words, the estimated conditional effect is statistically significant for levels of mindfulness less than 5.04 on the scale of mindfulness. The JN approach provides a continuous function of association between burnout and mind-wandering over all levels of mindfulness. All estimates are positive; however, at extremely high levels of mindfulness (specifically *W* > 5.04), the conditional direct effect of burnout on mind-wandering is not statistically significantly different from zero.

### 4.4. Putting It All Together

**Figure 5:**
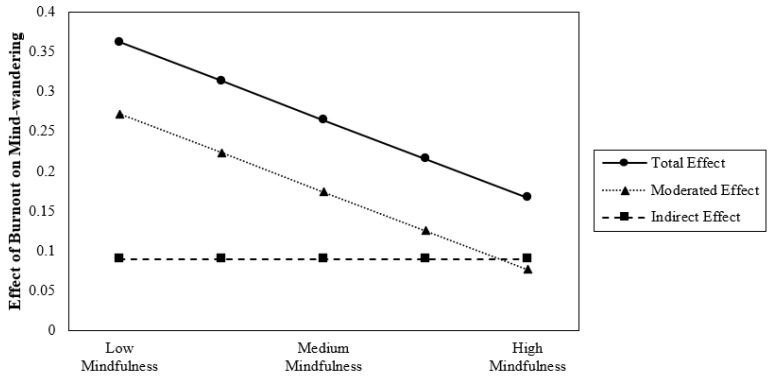
Putting it all together. *Note:* Dashed line is the indirect effect of burnout on mind-wandering through self-control. Dotted line is the moderated direct effect of burnout on mind-wandering. Black line is the total effect of burnout on mind-wandering, which is the sum of indirect and moderated direct effect.

Figure 5 shows the combined results of Hypothesis 1 through Hypothesis 3. In addition to an indirect positive effect of burnout on mind-wandering, mindfulness plays a critical role in burnout’s direct impact on mind-wandering. Burnout and mind-wandering demonstrate the weakest relationship when mindfulness is highest. In the same vein, burnout has the strongest relationship with mind-wandering when mindfulness is weakest. Said differently, at the highest levels of burnout, those with the highest levels of mindfulness have the least amount of mind-wandering and those with the lowest levels of mindfulness have the highest amount of interference.

## 5. Discussion

Drawing on self-regulatory strength models of self-control, this study investigated the “when” and “how” of burnout’s impact on attention. All hypotheses were supported. Results demonstrate that burnout exerts an indirect effect on mind-wandering through self-control. Burnout’s direct impact on attention is conditional on mindfulness. This was such that those with higher levels of mindfulness had a weaker relationship between burnout and attention while those with lower levels of mindfulness had a stronger relationship. In the following section, we discuss the theoretical and practical implications, limitations, and future directions that emerge from this research.

This study drives home the importance of depleted resources and their role in bringing on increased mind-wandering in the face of burnout. First, this research contributes to the literature on burnout by bringing to light a person-centric mechanism via attentional focus through which burnout may impact work and academic performance. We argue that burnout leads to internal energetic depletion that leads to a decline in self-control. The identification of self-control depletion as a key factor in leading to attention control failure highlights the importance of maintaining individuals’ internal energy and self-control resource availability. At a broader level, this study further confirms that strength and depletion theories may be an important explanatory mechanism for the negative outcomes that have been identified in past research on burnout (e.g., reduced performance, disinterestedness, and cognitive fatigue [1,99]). Additionally, this may open doors to an array of previously unexamined outcomes of burnout and provide a complimentary model of theoretical explanation to currently prevailing paradigms (e.g., job-demand resource model and conservation of resources model).

This study also highlights the importance of mindfulness as a buffering mechanism against the ill-effects of burnout on concentration. Previous research has highlighted the relationship between mindfulness and self-regulation [64,65]. This research adds to the body of knowledge and proposes new linkages in the association between self-regulation and attention by tying it into burnout. Previous research has demonstrated that mindfulness training leads to higher levels of attention, and other positive outcomes including gratitude [100], positive affect [101], short-term memory, and so on. Here, we highlight how burnout’s negative effects on attention may also be *attenuated* by mindfulness. Of particular interest is the finding using the JN approach; results hold up to relatively high levels of mindfulness. It appears differential mechanisms are at play for those with *extremely high levels* of mindfulness. Future research should endeavor to uncover the workings of extremely high levels of mindfulness. An appropriate sample for such an investigation would be individuals possessing exceptionally high levels of mindfulness (for instance, Buddhist monks whose lives are completely immersed in the mindful way of life).

This research has important implications for performance. By clarifying a potential pathway for why performance suffers in the case of burnout, it was found that distracting thoughts via mind-wandering and reduced self-control might be key reasons for why burnt-out individuals may find it difficult to concentrate. Future studies would do well to consider an experience sampling investigation to further parse out the mechanism behind burnout and mental interference.

### 5.1. Limitations

Due to the self-reported nature of this study, findings may be subject to common method variance (CMB [102]). Common method variance is a potential problem in psychological research. It refers to variance that occurs due to the measurement method, instead of the psychological constructs that the measures represent [103]. There are many sources of common method variance, including when data are collected from a single source in a self-report format, as in the present study. This may *inflate* correlational findings. However, it has been argued that self-report is the most appropriate form of measurement when the topic under investigation deals with inner psychological experiences and internal processes that may be unknown or less accurately known to even close others [104]. Mind-wandering, self-control, burnout, and mindfulness are intrinsic psychological processes that present greater validly when reported on by the participant, therefore necessitating self-report data if collected in non-laboratory or field settings. Thus, while correlations may be higher due to CMB, the overall trends in the patterns of the relationship would not be eliminated.

Given the cross-sectional nature of this study, it cannot make any claim on the causality of the observed relationships. All variables in this study are part of a cross-sectional dataset that was collected at a single time point from individual participants. Future research using temporally separate measures of the key variables using either longitudinal or experience sampling designs would be beneficial. The experience sampling method [105] may be particularly beneficial given the dynamic nature of human attention. Mind-wandering, by its very nature, is a dynamic aspect of the human experience and capturing the concomitant nature of the wandering mind in ecologically valid settings while people are at work or in schools can lend tremendous insight into its association with important predictor variables such as burnout. Multi-source data (such as organizational supervisor–subordinate dyads) would further do away with the limitations of self-reported data presented in the previous section.

For greater confidence in the causal nature of the observed relationships, an experimental paradigm may be beneficial. To explore causality, experimental designs may be explored for replicating these results. Mindfulness training as an intervention technique within an experimental setup can reveal its positive effects for managing and alleviating the symptoms of burnout.

Future research would also do well to examine other negative consequences of burnout from an ego-depletion standpoint, including behavioral ramifications at work, in academia, and in STEM to increase the generalizability of the results, particularly in organizational settings. As mentioned above, it is yet to be seen if the results of this investigation would generalize to work-settings. Investigations that replicate these findings in the context of the workplace would shed light on performance and work. Furthermore, it would be meaningful to investigate whether these relationships hold true across different demographic groups and individual difference factors. For instance, would certain STEM majors be more vulnerable to the depleting effects of burnout? Additionally, does this relationship change across the different years in college? Similarly, individual differences such as conscientiousness and neuroticism may play a protective or additionally negative role in these relationships that may account for group differences in the outcomes. Future research would do well to assess these phenomena.

Finally, the self-regulatory strength has received criticism and revision. However, despite the proposal of alternate models [106], different conceptualizations [107], and even the proposal that limited ego or willpower is a myth [62], the core tenet of depletion has not been extricated from our field [56]. Therefore, despite heavy criticism, the model still presents a meaningful theoretical basis to this study’s findings.

### 5.2. Practical Implications

This research opens the door to a variety of implications for academic and organizational interventions for both STEM and non-STEM settings. By clarifying a potential pathway for why performance suffers in the case of burnout, results suggest that distracting thoughts via mind-wandering and reduced self-control may be key to why burnt-out individuals find it difficult to concentrate [25]. To the extent that self-control resources underlie the negative consequences of burnout, the results of this study bring to light the importance of recovery and resource replenishment for supporting both organizationally relevant outcomes such as performance and individual health and well-being.

This is especially important in the case of STEM students and professionals. The STEM field is extremely demanding and requires a high amount of focus, dedication, hard work, long periods of study, cognitive labor, and continued mental exertion and high amounts of ensuing fatigue. It is well known that STEM professionals burn out at particularly alarming rates [77]. This study highlights the importance of taking steps to prevent the onset of burnout from the initial stages of the STEM pipeline. STEM educational bodies and STEM-based organizations may benefit from placing emphasis on recovery strategies.

This study reveals the relationship between burnout and outcomes related to academic achievement and performance. At the school and university-level, recovery strategies may be made a part of the educational curriculum to be taught to students from year one so that they can actively deal with burnout before it sets in. Evidence-based educational policies that focus on student wellness and improving student health may consider the inclusion of mindfulness training as part of district wellness policies and school-level practices for promoting students’ emotional and psychological health. Just like physical activity is part of many curricula and school policies for health promotion, short mindfulness training programs could be added to the students’ health promotion resources toolkit. Knowledge about burnout and its potential ill-effects and how to facilitate recovery and buffer against its ill effects can provide resiliency against especially demanding times in the lives of students, who may then be well equipped to carry these tools with them to their future careers and the world of work.

At the organizational level, employees should be encouraged to engage in active recovery strategies such as micro-breaks at work [108], psychological detachment [109], sleep [110], and self-affirmation [111]. Leadership in organizations should train managers to stay vigilant for employees in roles that may be more prone to burnout. Facilitating active recovery strategies for employees in positions that are susceptible to high stress and job-burnout can be beneficial for the organization. Similarly, fostering organizational cultures that value health and well-being and active recovery from work can encourage those in high-stress, high-demand jobs to unplug from work and pay attention to their psychological health. Those at-risk (such as in highly demanding roles) may be made aware of the possibility of burnout during organizational onboarding upon initial hiring.

Importantly, this research drives home the importance of mindfulness as an effective strategy to prevent mind-wandering in the face of burnout. While this study considered mindfulness as a trait-like variable, research has demonstrated that mindfulness is amenable to increases following training [69]. Extant research has demonstrated that mindfulness meditation can have a host of positive outcomes that have both physiological and psychological benefits. Organizations and academic bodies may use mindfulness training as an effective remedy against the cognitive ill-effects of burnout. Organization-supported initiatives that provide mindfulness training for employees may be beneficial. Similarly, educational bodies may provide mindfulness training for students during their education and training experiences. Future research may explore the benefits of other forms of meditative techniques such as breathing, chanting, and so on, on buffering the negative consequences of burnout.

Beyond educational institutions and academia, mindfulness meditation may be useful for any individual that may be suffering the adverse consequences of depletion and burnout. For instance, the implications of this study may be extended to those suffering from caregiver burnout, teacher burnout, and burnout as experienced by mental health and trauma professionals. Mindfulness meditation training may help protect against the negative consequences of burnout for society at a broader level.

## 6. Conclusions

Burnout influences mind-wandering indirectly through its effect on self-control. The impact of burnout on distracting thoughts is exacerbated in situations where the ability to observe one’s thoughts or stay in the moment is lower. This study highlights the role of burnout in different types of attentional processes for work and the buffering effect of mindfulness on this phenomenon. Findings of this investigation may contribute to the development of targeted interventions for STEM students, professionals, and other industries that may experience burnout along with concomitant attentional demands. Mindfulness training may be the key to reducing the performance-oriented ill-effects of burnout.

## Figures and Tables

**Table 1:** Means, standard deviations, reliabilities, and correlations for study variables.

Variables	*M*	SD	1	2	3	4
Burnout	3.59	1.01	0.90			
2. Self-control	3.36	0.51	−0.45 **	0.87		
3. Mindfulness	3.80	0.86	−0.38 **	0.43 **	0.89	
4. Mind-wandering	3.22	0.60	0.43 **	−0.45 **	−0.43 **	0.79

*Note*. *N* = 513. Alpha reliabilities are in italics and appear on the diagonal. *M*—mean, SD—standard deviation. ** *p* < 0.01.

**Table 2:** Hypothesis testing results.

Regression Results for the Total Effects Model					
**Effects**	**Coefficient**	**SE**	***t***	***p***	***Model R* ^2^**
*DV: Mind-wandering*					
Constant	2.29	0.09	25.82	0.000	
Burnout	0.26	0.02	10.88	0.000	0.19 ***
**Mediation Model**					
**Direct Effects**	**Coefficient**	**SE**	***t***	***p***	***Model R* ^2^**
*DV: Self-control*					
Constant	4.18	0.07	55.80	0.000	
Burnout	−0.23	0.02	−11.36	0.000	0.20 ***
*DV: Mind-wandering*					
Constant	3.87	0.23	17.10	0.000	
Burnout	0.17	0.03	6.83	0.000	
Self-control	−0.38	0.05	−7.54	0.000	0.27 ***
**Indirect Effect**	**Effect**	**Boot SE**	**Boot LLCI**		**Boot ULCI**
*Burnout on Mind-wandering*	0.09	0.015	0.059		0.118
**Moderation and Mediation Model**					
**Direct Effects**	**Coefficient**	**SE**	***t***	***p***	***Model R* ^2^**
*DV: Self-control*					
Constant	3.36	0.02	164.44	0.000	
Burnout	−0.23	0.02	−11.37	0.000	0.20 ***
*DV: Mind-wandering*					
Constant	4.26	0.18	24.11	0.000	
Burnout	0.14	0.03	5.32	0.000	
Self-control	−0.31	0.05	−5.94	0.000	
Mindfulness	−0.15	0.03	−5.09	0.000	
Burnout*Mindfulness	−0.05	0.02	−2.25	0.025	0.32 ***
**Conditional direct effects**	**Index**	**Boot SE**	**Boot LLCI**		**Boot ULCI**
Low mindfulness	0.174	0.030	0.116		0.233
Medium mindfulness	0.135	0.026	0.085		0.186
High mindfulness	0.093	0.032	0.029		0.157
**Indirect Effect**	**Effect**	**Boot SE**	**Boot LLCI**		**Boot ULCI**
*Burnout on Mind-wandering*	0.07	0.014	0.046		0.010

*Note*. *N* = 508. Effect size estimates are unstandardized coefficients. Moderator values of low, medium, and high are the 16th, 50th, and 84th percentiles. Mean centering used for product terms. *DV* is dependent variable. *SE* is standard error. *Boot* is 5000 bootstrap samples. *LLCI* is bias-corrected lower limit confidence interval. *ULCI* is bias-corrected upper limit confidence interval. *** is *p* < 0.001.

## Data Availability

Data may be available from the author within a certain time duration for academic research purposes only.
